# Association between increased mortality and bronchial fibroscopy in intensive care units and intermediate care units during COPD exacerbations: an analysis of the 2014 and 2015 National French Medical-based Information System Databases (PMSI)

**DOI:** 10.1186/s40560-021-00560-w

**Published:** 2021-06-15

**Authors:** Matthieu Amalric, Engi Ahmed, Boris Jung, Carey Suehs, Nicolas Molinari, Arnaud Bourdin, Jeremy Charriot

**Affiliations:** 1 Medical Intensive Care Unit, Montpellier University and Montpellier University Health Care Center, 34295 Montpellier, France; 2 Department of Respiratory Diseases, Montpellier University, CHU Montpellier, PhyMedExp, INSERM, CNRS, Montpellier, France; 3 Department of Medical Information, Montpellier University, Montpellier, France

**Keywords:** COPD, Bronchoscopy, Intensive care, Intermediate care, Mortality, PMSI, France

## Abstract

**Background:**

The course of chronic obstructive pulmonary disease (COPD) is punctuated by exacerbations, most often of infectious origin, responsible for many intensive care unit (ICU) and intermediate care unit (IMCU) admissions. Our objective was to study in-hospital mortality during severe COPD exacerbations in ICU and IMCU based on the performance of bronchoscopy.

**Methods:**

A retrospective analysis was carried out on stays in ICUs for COPD exacerbation from the French Programme for the Medicalisation of Information Systems databases for the years 2014 and 2015. Propensity score matching of stays made it possible to constitute two comparable groups on the factors of excess mortality described in the literature (age, sex, SAPS 2, type of admission and bronchial tumour).

**Results:**

We identified 14,491 stays for COPD exacerbation in ICUs, 2586 of which received a bronchoscopy. Mortality was significantly higher in the fibroscopy group (31.32% versus 19.8%). After propensity score matching, we found an excess of mortality in the intervention group (OR = 1.749 [1.516–2.017]) associated with a significantly longer length of stay. The main diagnoses associated with an increased risk of death were pulmonary embolism (OR = 3.251 [1.126–9.384]), bacterial pneumonia (OR = 1.906 [1.173–3.098]) and acute respiratory failure (OR = 1.840 [1.486–2.278]).

**Conclusions:**

Performing bronchoscopy during ICU hospitalisations for severe COPD exacerbations was associated with increased mortality. This increased mortality appears to be related to a bias in patient selection with a procedure reserved for patients with the adverse course.

**Supplementary Information:**

The online version contains supplementary material available at 10.1186/s40560-021-00560-w.

## Background

Chronic obstructive pulmonary disease (COPD) is a devastating disease characterised by an accelerated decline in respiratory function leading to disabling dyspnea and impaired quality of life [1, 2]. The prevalence of COPD, increasing with age [3], is estimated at 11.7% of the world’s population over 30 years of age [4]. It is now the third leading cause of death after cardiovascular diseases and stroke [5].

The trajectory of the disease may be punctuated by exacerbations, most often of infectious origin [3–6]. Hospitalisation for exacerbations is a significant adverse prognostic factor [6]. Admission in intensive care units for severe exacerbations is common. Funk et al. (2013) showed a 2.5% admission rate for COPD exacerbation among 194,453 stays in 87 intensive care units (ICUs) [7]. The intra-hospital mortality rate thus varies from 10% in inpatient departments to 23% in intensive care [8].

Bronchial fibroscopy is a technique routinely performed in ICUs. It is a fundamental examination in microbiological assessment of low-tract respiratory infections [9–11]. However, bronchoscopy with bronchoalveolar lavage leads to altered hematosis [12–14] and may induce transient lung function impairment with a decrease in FEV1 in patients with COPD [15]. Despite this procedure still being relatively safe [16, 17], the complication rate is significantly higher in patients with COPD and a FEV1 below 50% [18].

Our study follows on from a previous observational study that was based on the French National PMSI databases and aimed to study the evolution of hospitalisation characteristics for COPD exacerbation in France between 2007 and 2012. This study demonstrated an increase in deaths linked to exacerbation of infectious origin paradoxically associated with a decrease in the number of bronchial endoscopies [8]. In this context, the aim of our study here is to compare the mortality of patients hospitalised in IMCU and ICUs for severe COPD exacerbation, depending on whether or not a bronchial endoscopy is carried out.

The objective of our study was to study in-hospital mortality during severe COPD exacerbations in the intermediate care unit (IMCU) and intensive care unit (ICU) based on the performance of bronchoscopy.

## Materials and methods

### Definition of the population

We conducted a retrospective observational study based on the analysis of data from the National French “Programme for the Medicalisation of Information Systems” (PMSI). This database, covering all French private and public health care institutions, contains medico-economic information on admissions.

The study focused on COPD exacerbation admissions in IMCU and ICUs over a 24-month period (2014–2015). The selection algorithm for hospital stays, based on the international classification of diseases (ICD-10), is given in Supplemental Digital Content – Table 1. This has been validated by two previously published studies [8, 19]. Patients with codes for chronic airway conditions other than COPD, such as asthma, bronchiectasis or restrictive chronic respiratory failure, were excluded. The ICD-10 codes used are referenced in Supplemental Digital Content – Table 2.

Given the study is based on the analysis of an anonymous national database, it falls under the law on January 6, 1978, revised on January 26, 2016 (law no. 2016-41). As a result, no consent was required.

### Practice survey among intensivists

In order to explain the results obtained, we performed a local practice survey among senior intensivists in the University Hospital of Montpellier about their use of bronchoscopy for severe acute exacerbations of COPD.

### Statistical analysis

Qualitative variables are described by frequency, and quantitative variables by median and inter-quartiles (25th and 75th percentiles). Comparison between groups was performed using the non-parametric Wilcoxon-Mann-Whitney test for quantitative variables and the chi-square test for qualitative variables. The significance threshold was set at 5% for the different tests used. The non-Gaussian character of the distribution of all quantitative variables was demonstrated by the Kolmogorov-Smirnov test.

Several factors are predictive of excess hospital mortality during COPD exacerbations: age, male sex, number of previous hospitalisations for exacerbations, exacerbation severity, association with other comorbidities (in particular with pulmonary neoplasia) or COPD severity [19, 20]. A propensity score was calculated in order to balance the groups between these parameters. The variables used to calculate the propensity score were age, sex, type of hospitalisation (IMCU or ICU), bronchial cancer and the Simplified Acute Physiology Score (SAPS) 2 (in the squared-form in order to detect small differences in SAPS 2). We then created two balanced groups on this parameter with a 1:1 match. The balance of the groups, as well as the mortality analysis, was tested using the statistical methods described above.

The extraction of the PMSI data and statistical analyses was carried out with the help of the Department of Medical Information team at the University Hospital Centre of Montpellier.

## Results

### Population characteristics

We identified 14,491 stays for COPD exacerbation admitted to either IMCUs or ICUs in France over a 24-month period: 6873 in 2014 and 7618 in 2015. Only 2586 patients received bronchial endoscopy, i.e. 1268 in 2014 and 1318 in 2015.

The characteristics of the population are provided in Table 1. Patients receiving fibroscopy were younger (p < 0.001), had a sex ratio favouring male (p < 0.001), had a higher median length of stay (MLS) (p < 0.001), had a higher rate of bronchial tumours (p < 0.001), had more severe exacerbations with a higher median SAPS 2 score and intensive care admission rate (p < 0.001). The mortality rate was higher in the fibroscopy group with 31.32% versus 19.08% (p<0.001) for the without fibroscopy group.

**Table 1:** Characteristics of patients admitted to resuscitation and ICUs for COPD exacerbation in France over 24 months (2014-2015)

Patient characteristics	Without fibroscopy n = 11905	Fibroscopy n = 2586	p
Median age (years)	74 [63 ; 82]	68 [59 ; 77]	< 0.001
Male sex	7490 (62.91%)	1907 (73.74%)	< 0.001
MLS (days)	13 [8 ; 21]	25 [15 ; 41]	< 0.001
Median SAPS 2	23 [0 ; 40]	28 [0 ; 45]	< 0.001
ICU admission	6857 (57.60%)	2113 (81.71%)	< 0.001
Bronchial tumour	336 (2.82%)	216 (8.35%)	< 0.001
Deaths	2272 (19.08%)	810 (31.32%)	< 0.001

Values are numbers (%) or medians [Q25, Q75]

p-value for trend test; *MLS* median length of stay, *ICU* intensive care unit

### Seasonality

**Fig. 1:**
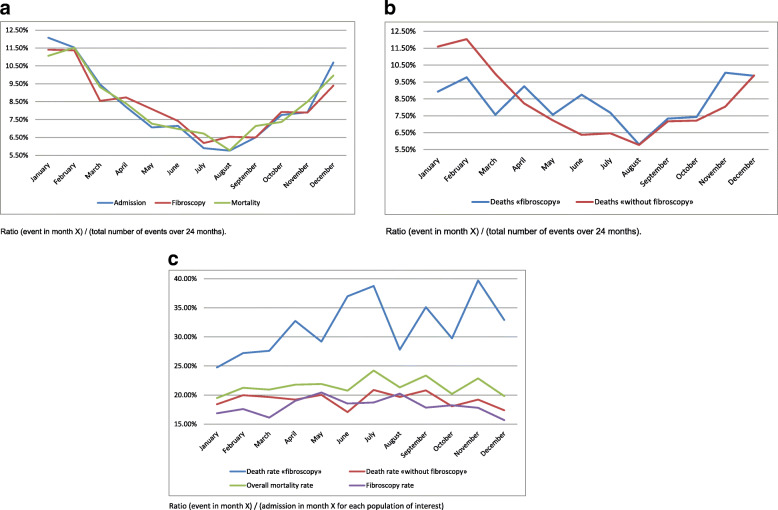
**a** Annual distribution of average admissions, fibroscopic procedures and mortality for 2014 and 2015. Ratio (event in month X)/(total number of events over 24 months). **b** Annual distribution of average deaths in each population for 2014 and 2015. Ratio (event in month X)/(total number of events over 24 months). **c** Average annual change in monthly mortality and fibroscopy rates for 2014 and 2015. Ratio (event in month X)/(admission in month X for each population of interest)

Figure 1a shows the annual distribution of admissions, mortality and fibroscopic procedures averaged for 2014 and 2015. It is defined by the ratio (event in month X)/(total number of events over 24 months). We identified a seasonal variation with an increase in intermediate care and intensive care admissions, the number of bronchial fibroscopies and mortality during the winter period. The study of annual mortality distribution for both groups is shown in Fig. 1b. The seasonal mortality pattern in the non-fibroscopy group had a similar pattern to the mortality rate in the overall population described in Fig. 1a, unlike the fibroscopy group in which the seasonal variability appeared to be less pronounced.

Figure 1c shows the average change in monthly mortality and fibroscopy rates for 2014 and 2015. These rates are defined by the ratio (event in month X)/(admission in month X for each population of interest). By relating each event to the number of patients admitted each month for each population of interest, we identified several additional results. Firstly, the fibroscopy rate was relatively stable over the year, with a trend towards a decrease in use of this examination during the winter months. Secondly, the mortality rate in the without fibroscopy group remained stable throughout the year at between 17 and 20.8%. In the fibroscopy group, the mortality rate ranged from 24.7 to 39.7%, thus remaining higher than in the non-fibroscopy group throughout the year. In addition, there was an increase in the mortality rate during the summer months.

### Propensity score

Among the 2586 stays receiving bronchial endoscopy, at least one data required for calculating the propensity score was missing for 573 stays. Given the group of patients receiving bronchial fibroscopy was in the minority, we obtained two groups each of 2103 stays. The groups were comparable on all the criteria defined for calculating the propensity score as shown in Table 2. The median length of stay was longer in the fibroscopy group (p<0.001). Despite the comparability of the two groups regarding predictive factors of excess mortality, the death rate remained higher in the bronchial endoscopy group at 29.62% versus 19.40% (p<0.001) for the non-fibroscopy group, representing an odds ratio of (1.749 [1.516–2.017]). Twenty senior intensivists over 57 responded to the practice survey (response rate = 35.1%). Thirty percent reported to perform a bronchoscopy systematically at admission versus 85% only in case of clinical worsening (Supplemental Digital Content – Table 5).

**Table 2:** Patient characteristics after propensity score matching

Patient characteritics	Without fibroscopy n = 2103	Fibroscopy n = 2103	p
Median age (years)	68 [59 ; 77]	67 [59 ; 76]	0.374
Male sex	1590 (75.61%)	1555 (73.94%)	0.214
Median SAPS 2	27 [0 ; 43]	28 [0 ; 45]	0.194
ICU admission	1756 (83.50%)	1735 (82.50%)	0.389
Bronchial tumour	152 (7.23%)	176 (8.37%)	0.167
Deaths	408 (19.40%)	623 (29.62%)	< 0.001
MLS (days)	14 [8 ; 23]	24 [14 ; 40]	< 0.001

Values are numbers (%) or medians [Q25, Q75]

p-value for trend test; *MLS* median length of stay, *ICU* intensive care unit

### Primary diagnosis (PD)

Supplemental Digital Content – Table 3 lists all the PDs scored for the two groups of 2103 patients from the propensity scoring. The PDs associated with a significantly lower fibroscopy rate were pulmonary embolism (p<0.01), heart failure (p<0.01), COPD (p<0.01) and acute bronchitis (p<0.01). PDs associated with a significantly higher rate of fibroscopy was acute respiratory distress syndrome (ARDS) (p<0.01), lung abscess with pneumonia (p<0.01) and unclassified bacterial pneumonia (p<0.01).

Supplemental Digital Content – Table 4 lists the PDs of deceased patients extracted from the groups derived from the propensity score, i.e. 1031 stays corresponding to 408 and 623 stays in the without fibroscopy and fibroscopy groups, respectively. Table 3 gives the odds ratios for mortality regarding the different PDs depending on whether or not a fibroscopic procedure was performed. There was increased mortality in the fibroscopy group with PDs of pulmonary embolism (p=0.03), unclassified bacterial pneumonia (p<0.01), acute respiratory failure (p<0.01) and COPD (p<0.01).

**Table 3:** Odds ratios for mortality in the groups derived from propensity scoring

Outcomes	OR	p
Pulmonary embolism (I26)	3.251 [1.126 – 9.384]	0.0293
Cardiac failure (I500)	1.595 [0.909 – 2.801]	0.1037
Identified influenza virus (J10)	2.053 [0.192 – 21.97]	0.5521
*Pneumonia due to Streptococcus pneumoniae* (J13)	0.735 [0.186 – 2.908]	0.6605
Bacterial pneumonia, unspecified (J15)	1.906 [1.173 – 3.098]	0.0092
Pneumonia in bacterial diseases (J17)	1.682 [0.929 – 3.044]	0.0861
Pneumonia, unspecified organism (J18)	1.743 [1.197 – 2.539]	0.0038
Acute respiratory distress syndrome (J80)	1.349 [0.863 – 2.110]	0.1884
Pneumothorax (J93)	0.208 [0.015 – 2.854]	0.2401
Acute respiratory failure (J96)	1.840 [1.486 – 2.278]	< 0.001

*p* p-value for trend test, *OR* odds ratio

## Discussion

The analysis of 14,491 stays for COPD exacerbation in intermediate care unit and ICUs over a 24-month period from the national PMSI database highlights three main findings.

Firstly, only 17.8% of patients received a fibroscopic procedure. This result is explained by the lack of guidelines demanding invasive microbiological assessment in first-line treatment for severe COPD exacerbations, with antibiotic therapy being initiated probabilistically after non-invasive sampling [21]. However, there is a significant proportion of multidrug-resistant bacteria in these patients that could be responsible for failure of the initial antibiotic challenge, resulting in increased mortality [22]. Although not universally accepted as the gold standard, bronchoscopy shows great performance in the microbiological assessment of lower respiratory tract infections (LRTIs). In critically ill patients, bronchoalveolar lavage fluid showed the higher predictive value for the microbiological diagnosis of ventilator-acquired pneumonia (VAP) [23]. In the same setting, invasive procedures to obtain microbiological diagnosis in LRTIs were even associated with fewer deaths at day 14 [24], although this result had been mitigated in the study by Luna et al. [25] in which, despite the high accuracy of bronchoscopy to detect VAP, this information becomes available too late to positively influence survival.

Secondly, despite the procedure being recognised as relatively safe [16–18], we found a higher mortality rate in the group of patients having received bronchial endoscopy (31.32%) compared to without (19.08%) (p < 0.001). This result was confirmed following analysis by propensity score matching that balanced the two groups on the excess mortality factors identified in the literature [19, 20]. It also appears that patients who received fibroscopy had a prolonged length of stay. This leads to the hypothesis that fibroscopy is reserved for a selected population which develops unfavourably during the intensive care stay. However, the methodology used does not allow us to conclude this. This result is consistent with those obtained in a study of ventilator-acquired pneumonia where invasive microbiological sampling by fibroscopy did not lead to an increase in mortality, reduction in the length of stay in intensive care or reduction in the length of time spent on mechanical ventilation [26].

Thirdly, it is recognised that seasonality is a factor affecting COPD exacerbations with increased incidence, hospitalisations and mortality during the winter months. Our data are consistent with the current literature on these points [27, 28]. Mortality rates in the overall population and the fibroscopy rate remain relatively stable throughout the year. The preferential distribution of endoscopy numbers and deaths thus appears to be a number-related effect associated to an increased incidence of COPD exacerbations during the winter months. In contrast to the no endoscopy group, where the mortality rate remains relatively stable throughout the year, the mortality rate in the endoscopy group increases sharply during the summer months. The cause of this excess summer mortality is, however, difficult to explain.

Finally, the PD is defined as “the reason driving the bulk of the medical and care effort during hospitalisation”, and we have thus assimilated it to the etiological diagnosis of the exacerbation. The majority of the numbers (2887 in Supplemental Digital Content – Table 3 and 750 in Supplemental Digital Content – Table 4) are classified as acute respiratory failure, respiratory distress syndrome or COPD, making an etiologic diagnosis of the exacerbation impossible. The methodology of our study does not allow precise determination of the indications of the endoscopic examinations performed and the cause of mortality therefore requires additional study.

The main strengths of our study are the large number of stays analysed and the completeness of the database. The latter is largely related to the legal obligations of PMSI coding of hospital stays at discharge by the medical team combined with quality control by health insurance doctors. In addition, patient selection bias is limited by a standardised algorithm validated by two previously published studies [8, 29].

Several weaknesses are associated with studies such as ours. Firstly, there is no chronology in the coding of medical acts. Although it is recognised that recourse to the use of invasive mechanical ventilation is associated with an unfavourable prognosis [30, 31], we decided not to consider this parameter in our analysis. Indeed, it was impossible to distinguish between patients according to their ventilation modality at the time the fibroscopic procedure was performed. Secondly, even if the use of propensity scoring has strongly developed in medical literature in recent years [29], it seems insufficient to completely eliminate heterogeneity in the results between different studies.

## Conclusion

Our study shows increased mortality among patients receiving bronchial fibroscopy during hospitalisation in intermediate care or intensive care for COPD exacerbation. This excess mortality seems enigmatic for a procedure that not only has a good tolerance well documented in the literature, but is also a reference for microbiological assessment of respiratory infections. The most likely hypothesis to explain this result is that this examination is used preferentially in specific indications, for a selected and more severe population with a less favourable prognosis independent of the absence of fibroscopy.

## Supplementary Information

**Additional file 1: Supplemental Digital Content – Table 1**. Definition of hospital stays in resuscitation / ICUs for COPD exacerbation based on ICD-10 codes.

**Additional file 2: Supplemental Digital Content – Table 2.** Definition of ICD-10 Codes.

**Additional file 3: Supplemental Digital Content – Table 3.** PDs of patients in the groups derived from propensity scoring.

**Additional file 4: Supplemental Digital Content – Table 4.** PDs of deceased patients in the propensity score groups.

**Additional file 5: Supplemental - Table 5.** Practice Survey among intensivists about their use of bronchoscopy during an acute exacerbation of COPD.

## Data Availability

The datasets used and/or analysed during the current study are available from the corresponding author on reasonable request.

## Abbreviations

COPD: Chronic obstructive pulmonary disease
FEV1: Forced expiratory volume in the 1st second
ICU: Intensive care unit
IMCU: Intermediate care unit
ICD-10: International Classification of Diseases
MLS: Median length of stay
OR: Odds ratio
PD: Primary diagnosis
PMSI: Programme de médicalisation des systèmes d'information (National French Medical-based Information System Database)
SAPS: Score Simplified Acute Physiology Score
