# Supplementary material for: Association between increased mortality and bronchial fibroscopy in intensive care units and intermediate care units during COPD exacerbations: an analysis of the 2014 and 2015 National French Medical-based Information System Databases (PMSI)
Source: J Intensive Care. 2021 Jun 15;9:45. doi: 10.1186/s40560-021-00560-w (PMC8205318; doi:10.1186/s40560-021-00560-w)
Supplement: Supplementary file 1 — Additional file 1: Supplemental Digital Content – Table 1. Definition of hospital stays in resuscitation / ICUs for COPD exacerbation based on ICD-10 codes. [file 40560_2021_560_MOESM1_ESM.docx]

Supplemental Digital Content – Table 1: Definition of hospital stays in resuscitation / ICUs for COPD exacerbation based on ICD-10 codes.

| Primary Diagnosis | Associated Diagnosis | Remarks |
| --- | --- | --- |
| J41 or J42 or J43 or J44 or J961+0 | **J80** or **J960** | With CCAM code validating intensive care fee (**YYYY015** or **YYYY020**)  Exclusion of:  **J45** and **J46** and **J47** and **J95** and **J98** and **J961+1** |
| or | |  |
| I26 or I500 or I270.0 or J13 or J14 or J18 or J20 or J40 or J93 or J100 or J110 or J120 or J121 or J122 or J123 or J128 or J150 or J151 or J152 or J153 or J154 or J155 or J156 or J157 or J158 or J159 or J160 or J168 or J170 or J851 or U049 | **J41** or **J42** or **J43** or **J44** or **J961+0**  and  **J80** or **J960** |  |

**CCAM: Common classification of Medical Acts;** **YYYY015: Intensive care fee level A; YYYY020: Intensive care fee level B**
