# Supplementary material for: Association between increased mortality and bronchial fibroscopy in intensive care units and intermediate care units during COPD exacerbations: an analysis of the 2014 and 2015 National French Medical-based Information System Databases (PMSI)
Source: J Intensive Care. 2021 Jun 15;9:45. doi: 10.1186/s40560-021-00560-w (PMC8205318; doi:10.1186/s40560-021-00560-w)
Supplement: Supplementary file 2 — Additional file 2: Supplemental Digital Content – Table 2. Definition of ICD-10 Codes. [file 40560_2021_560_MOESM2_ESM.docx]

**Supplemental Digital Content – Table 2: Definition of ICD-10 Codes**

| ICD-10 | Description |
| --- | --- |
| J41 | Mucopurulent simple chronic bronchitis |
| J42 | Chronic bronchitis, unspecified |
| J43 | Emphysema |
| J44 | Other chronic obstructive pulmonary diseases |
| J45 | Asthma |
| J46 | Status asthmaticus |
| J47 | Bronchectasie |
| J80 | Acute respiratory distress syndrome |
| J95 | Respiratory disorders after a diagnostic and therapeutic procedure, not elsewhere classified |
| J98 | Other respiratory disorders |
| J960 | Acute respiratory failure |
| J961+0 | Chronic obstructive pulmonary failure |
| J961+1 | Chronic restrictive respiratory failure |
| I26 | Pulmonary embolism |
| I500 | Congestive heart failure |
| I270 | Pulmonary hypertension |
| J13 | Pneumonia due to Streptococcus pneumoniae (J13) |
| J14 | Haemophilus influenzae pneumonia (J14) |
| J18 | Pneumonia, unspecified organism (J18) |
| J20 | Acute bronchitis (J20) |
| J40 | Bronchitis |
| J93 | Pneumothorax |
| J100 | Influenza with pneumonia, seasonal influenza virus identified |
| J110 | Influenza with pneumonia, unidentified virus |
| J120 | Adenoviral pneumonia |
| J121 | Respiratory syncytial virus pneumonia |
| J122 | Parainfluenza virus pneumonia |
| J123 | Human metapneumovirus pneumonia |
| J128 | Other viral pneumonias |
| J150 | Pneumonia due to Klebsiella pneumoniae |
| J151 | Pneumonia due to Pseudomonas |
| J152 | Pneumonia due to staphylococcus |
| J153 | Pneumonia due to staphylococcus, group B |
| J154 | Pneumonia due to other streptococci |
| J155 | Pneumonia due to Escherichia coli |
| J156 | Pneumonia due to other gram-negative bacteria |
| J157 | Pneumonia due to Mycoplasma pneumoniae |
| J158 | Other bacterial pneumonias |
| J159 | Bacterial pneumonia, unspecified |
| J168 | Pneumonia due to other infectious organisms |
| J170 | Pneumonia during bacterial diseases classified elsewhere |
| J851 | Lung abscess with pneumonia |
| U049 | Severe acute respiratory syndrome |
