# Supplementary material for: Association between increased mortality and bronchial fibroscopy in intensive care units and intermediate care units during COPD exacerbations: an analysis of the 2014 and 2015 National French Medical-based Information System Databases (PMSI)
Source: J Intensive Care. 2021 Jun 15;9:45. doi: 10.1186/s40560-021-00560-w (PMC8205318; doi:10.1186/s40560-021-00560-w)
Supplement: Supplementary file 3 — Additional file 3: Supplemental Digital Content – Table 3. PDs of patients in the groups derived from propensity scoring. [file 40560_2021_560_MOESM3_ESM.docx]

Supplemental Digital Content – Table 3: PDs of patients in the groups derived from propensity scoring.

|  | Without fibroscopy  n = 2103 | Fibroscopy  n = 2103 | p |
| --- | --- | --- | --- |
| Pulmonary embolism (I26) | 73 (3.47) | 39 (1.85) | 0.001 |
| Cardiac failure (I500) | 190 (9.03) | 88 (4.18) | < 0.001 |
| Identified influenza virus (J10) | 14 (0.67) | 22 (1.05) | 0.1805 |
| Influenza due to unidentified influenza virus (J11) | 4 (0.19) | 3 (0.14) | 0.7052 |
| Viral pneumonia (J12) | 8 (0.38) | 5 (0.24) | 0.4046 |
| Pneumonia due to Streptococcus pneumoniae (J13) | 50 (2.4) | 53 (2.52) | 0.7647 |
| Haemophilus influenzae pneumonia (J14) | 11 (0.52) | 17 (0.81) | 0.2552 |
| Bacterial pneumonia, unspecified (J15) | 174 (8.27) | 250 (11.89) | < 0.001 |
| Pneumonia due to other infectious organisms (J16) | 2 (0.09) | 5 (0.24) | 0.2564 |
| Pneumonia in bacterial diseases (J17) | 5 (0.23) | 2 (0.09) | 0.2564 |
| Pneumonia, unspecified organism (J18) | 126 (5.99) | 141 (6.70) | 0.3428 |
| Acute bronchitis (J20) | 14 (0.67) | 3 (0.14) | 0.0075 |
| Simple and mucopurulent chronic bronchitis (J41) | 3 (0.14) | 0 | 0.0831 |
| Emphysema (J43) | 1 (0.05) | 3 (0.14) | 0.3170 |
| Other chronic obstructive pulmonary diseases (J44) | 389 (18.50) | 278 (13.22) | < 0.001 |
| Acute respiratory distress syndrome (J80) | 134 (6.37) | 244 (11.60) | < 0.001 |
| Abscess of lung with pneumonia (J85) | 1 (0.05) | 16 (7.61) | < 0.001 |
| Pneumothorax (J93) | 7 (0.33) | 13 (0.62) | 0.1786 |
| Acute respiratory failure (J96) | 895 (42.56) | 920 (43.75) | 0.4363 |
| Severe acute respiratory syndrome (U04) | 2 (0.09) | 1 (0.05) | 0.5635 |

Data are summarized as n (%)

Definition of abbreviations: p: p-value for trend test
