# Supplementary material for: Association between increased mortality and bronchial fibroscopy in intensive care units and intermediate care units during COPD exacerbations: an analysis of the 2014 and 2015 National French Medical-based Information System Databases (PMSI)
Source: J Intensive Care. 2021 Jun 15;9:45. doi: 10.1186/s40560-021-00560-w (PMC8205318; doi:10.1186/s40560-021-00560-w)
Supplement: Supplementary file 4 — Additional file 4: Supplemental Digital Content – Table 4. PDs of deceased patients in the propensity score groups. [file 40560_2021_560_MOESM4_ESM.docx]

Supplemental Digital Content – Table 4: PDs of deceased patients in the propensity score groups.

|  | Without fibroscopy  n = 408 | Fibroscopy  n = 623 | p |
| --- | --- | --- | --- |
| Pulmonary embolism (I26) | 7 (1.71) | 10 (1.60) | 0.892 |
| Cardiac failure (I500) | 43 (10.54) | 28 (4.49) | < 0.001 |
| Identified influenza virus (J10) | 1 (0.25) | 3 (0.48) | 1.000 |
| Influenza due to unidentified influenza virus (J11) | 1 (0.25) | 0 | 0.396 |
| Viral pneumonia (J12) | 3 (0.73) | 0 | 0.062 |
| Pneumonia due to Streptococcus pneumoniae (J13) | 5 (1.22) | 4 (0.64) | 0.330 |
| Haemophilus influenzae pneumonia (J14) | 0 | 3 (0.48) | 0.282 |
| Bacterial pneumonia, unspecified (J15) | 29 (7.11) | 69 (11.07) | 0.034 |
| Pneumonia due to other infectious organisms (J16) | 1 (0.25) | 2 (0.32) | 1.000 |
| Pneumonia in bacterial diseases (J17) | 1 (0.25) | 0 | 0.396 |
| Pneumonia, unspecified organism (J18) | 22 (5.39) | 37 (5.94) | 0.712 |
| Acute bronchitis (J20) | 2 (0.49) | 0 | 0.156 |
| Simple and mucopurulent chronic bronchitis (J41) | 1 (0.25) | 0 | 0.396 |
| Other chronic obstructive pulmonary diseases (J44) | 66 (16.18) | 73 (11.72) | 0.040 |
| Acute respiratory distress syndrome (J80) | 42 (10.29) | 93 (14.93) | 0.031 |
| Abscess of lung with pneumonia (J85) | 0 | 6 (0.96) | 0.087 |
| Pneumothorax (J93) | 2 (0.49) | 1 (0.16) | 0.566 |
| Acute respiratory failure (J96) | 182 (44.61) | 294 (47.19) | 0.416 |

Data are summarized as n (%)

Definition of abbreviations: p: p-value for trend test
