# Supplementary material for: Association between increased mortality and bronchial fibroscopy in intensive care units and intermediate care units during COPD exacerbations: an analysis of the 2014 and 2015 National French Medical-based Information System Databases (PMSI)
Source: J Intensive Care. 2021 Jun 15;9:45. doi: 10.1186/s40560-021-00560-w (PMC8205318; doi:10.1186/s40560-021-00560-w)
Supplement: Supplementary file 5 — Additional file 5: Supplemental - Table 5. Practice Survey among intensivists about their use of bronchoscopy during an acute exacerbation of COPD. [file 40560_2021_560_MOESM5_ESM.docx]

Supplemental - Table 5: Practice Survey among intensivists about their use of bronchoscopy during an acute exacerbation of COPD

|  | Number of responses (n=57) |
| --- | --- |
| Type of Unit |  |
| ICU | 19 (47.5%) |
| IMCU | 1 (5.2%) |
| Indication for bronchoscopy |  |
| Systematically at admission | 6 (30%) |
| Clinical worsening | 17 (85%) |
| Parenchymal opacities suspected of malignancy | 20 (100%) |
| Ventilator Acquired Pneumonia | 12 (60%) |
| Other | 3 (15%) |
| Sampling method |  |
| Bronchial Brushing | 0 |
| Bronchoalveolar lavage fluid | 17 (85%) |
| Guided bronchial aspirate | 9 (45%) |
| Orotracheal intubation specifically to perform the procedure | 2 (10%) |
| Bronchoscopy in patients with Spontaneous Breathing | 11 (55%) |
| Pulmonologist contacted for the procedure |  |
| Patients with spontaneous breathing | 19 (100%)* |
| All procedures | 0 |
| Therapeutical change induced by bronchoscopy |  |
| Always | 0 |
| Often | 14 (70%) |
| Rarely | 6 (30%) |
| Never | 0 |

ICU: Intensive Care Unit; IMCU: Intermediate Care Unit; * All intensivists from ICU reported calling a pulmonologist for bronchoscopy in patients with spontaneous breathing
